# Associations between bullying and risk for eating disorders in adolescents

**DOI:** 10.1590/0034-7167-2022-0643

**Published:** 2023-11-27

**Authors:** Priscilla dos Reis Oliveira, Marta Angélica Iossi Silva, Wanderlei Abadio de Oliveira, André Vilela Komatsu, Marisa Afonso de Andrade Brunherotti, Rafaela Rosário, Jorge Luiz da Silva

**Affiliations:** I Universidade de Franca. Franca, São Paulo, Brazil; II Universidade de São Paulo. Ribeirão Preto, São Paulo, Brazil; III Pontifícia Universidade Católica de Campinas. Campinas, São Paulo, Brazil; IV Universidade de São Paulo. São Paulo, São Paulo, Brazil; V Universidade do Minho. Braga, Braga, Portugal

**Keywords:** Bullying, Feeding and Eating Disorders, Adolescent, Risk, Association, Acoso Escolar, Trastornos de Alimentación y de la Ingestión de Alimentos, Adolescente, Riesgo, Asociación, Bullying, Transtornos da Alimentação e da Ingestão de Alimentos, Adolescente, Risco, Associação

## Abstract

**Objectives:**

to analyze the associations between bullying participation profiles (victims, bullies, and bully-victims) and the risk for eating disorders in adolescents.

**Methods:**

a cross-sectional study was conducted with 491 students, aged 10 to 18 years. Data were collected through the application of the Peer Victimization and Aggression Scale and the Eating Attitudes Test, and were statistically analyzed using analysis of variance (ANOVA) and Spearman correlation.

**Results:**

the risk for eating disorders was higher for the victim profile, both for boys and girls. For both sexes, physical victimization, verbal victimization, and relational victimization were significantly associated with variables related to the risk for eating disorders. For boys, there were also significant associations related to aggression.

**Conclusions:**

student victims, especially boys, are more vulnerable to the consequences of bullying in relation to the risk for eating disorders.

## RESUMO

**Objetivos:**

analisar as associações entre o perfil de participação no *bullying* (vítimas, agressores e vítimas-agressoras) e o risco de transtornos alimentares em adolescentes.

**Métodos:**

estudo transversal realizado com 491 estudantes, com idades entre 10 e 18 anos. Os dados foram coletados por meio da aplicação da Escala de Vitimização e Agressão entre Pares e do *Eating Attitudes Test*, e foram analisados estatisticamente por meio de análises de variância (ANOVA) e correlação de Spearman.

**Resultados:**

o risco de transtornos alimentares foi maior para o perfil de vítima, tanto para meninos quanto para meninas. Para ambos os sexos, a vitimização física, a vitimização verbal e a vitimização relacional associaram-se significativamente às variáveis de risco de transtornos alimentares. Para os meninos, também houve associações significativas relacionadas à agressão.

**Conclusões:**

os estudantes vítimas, especialmente os meninos, são mais vulneráveis às consequências do *bullying* em relação ao risco de transtornos alimentares.

## RESUMEN

**Objetivos:**

analizar las asociaciones entre los perfiles de participación en el acoso escolar (víctimas, agresores y víctimas-agresores) y el riesgo de trastornos alimentarios en adolescentes.

**Métodos:**

estudio transversal con 491 estudiantes de 10 a 18 años. Los datos se recopilaron mediante la aplicación de la Escala de Victimización y Agresión entre Pares y el Test de Actitudes Alimentarias, y se analizaron estadísticamente mediante análisis de varianza (ANOVA) y correlación de Spearman.

**Resultados:**

el riesgo de trastornos alimentarios fue mayor en el perfil de víctima, tanto en niños como en niñas. Tanto para ambos sexos, la victimización física, la victimización verbal y la victimización relacional se asociaron significativamente con variables relacionadas con el riesgo de trastornos alimentarios. En el caso de los niños, también se observaron asociaciones significativas relacionadas con la agresión.

**Conclusiones:**

los estudiantes que son víctimas, especialmente los niños, son más vulnerables a las consecuencias del acoso escolar en relación con el riesgo de trastornos alimentarios.

## INTRODUCTION

Bullying occurs when one or more individuals intentionally and repeatedly harm others in interactions characterized by a power imbalance^(1)^. It is a form of peer violence that can happen in various settings, but is particularly prevalent in schools^(2)^. In school environments, students can engage in aggressive behavior (bullies), become targets of such behavior (victims), or both perpetrate and experience violence simultaneously (bully-victims)^(3)^. The aggression can be physical (hitting, pushing, kicking, etc.), verbal (name-calling, mocking, laughing, for example), or relational (threatening, socially isolating a peer, among others)^(4)^.

The National School Health Survey (PeNSE) identified a prevalence rate of 28% for involvement in bullying situations in a nationally representative sample of ninth-grade students in Brazil^(5)^. Due to its high prevalence and the negative consequences, it has on academic performance and the psychosocial development of children and adolescents, bullying is considered a public health issue^(6-7)^. Some of its consequences include anxiety, low self-esteem, depression, poor academic achievement, phobias, and suicide^(8)^.

Bullying can also pose a risk for eating disorders^(9)^, which are psychiatric conditions characterized by disruptions in eating behavior. The most extensively studied eating disorders are anorexia nervosa, bulimia nervosa, and binge-eating disorder^(10)^. These disorders occur more frequently in women, especially in adolescents and young adults. Anorexia nervosa involves an excessive fear of gaining weight, accompanied by a severe distortion of body image. Bulimia nervosa entails episodes of binge eating followed by feelings of guilt, leading to compensatory behaviors such as induced vomiting. Binge-eating disorder occurs when a person consumes large quantities of food within a short period, typically two hours, even when not hungry^(11)^.

Eating disorders can be caused and perpetuated by psychological, metabolic, biological, familial, school-related, or sociocultural factors^(12)^. Specifically, regarding school factors, bullying related to physical appearance can trigger issues related to eating and concerns about weight control^(13)^. However, there is evidence that not only appearance-related aggression influences the desire to modify body size and shape, but that any form of bullying is associated with eating problems^(14)^. For instance, a study found that adolescents aged 11 to 16 who experienced physical, relational, or cyberbullying expressed concerns about weight loss, irrespective of their actual weight^(15)^.

As individuals involved in bullying can experience eating-related issues, their physical and mental health can be compromised for extended periods, as most adolescents with eating disorders continue to exhibit symptoms even ten years later^(16)^. Considering that eating disorders often emerge during adolescence, a period when bullying is also more prevalent, it is important to conduct studies involving adolescents to gather knowledge that can inform policies and programs aimed at preventing or reducing school bullying. In this regard, although there are studies linking bullying to symptoms of eating disorders, few explore the differences among participant profiles to determine whether the associations are stronger for victims, bullies, or bully-victims^(17)^.

## OBJECTIVES

To analyze the associations between the profiles of bullying participation (victims, bullies, and bully-victims) and the risk of eating disorders in adolescents.

## METHODS

### Ethical aspects

Data collection began after obtaining approval from the Research Ethics Committee. The research was authorized by the school administration of a public school, and parental/guardian authorization was obtained through the signing of an Informed Consent Form (ICF). The adolescents also signed an Assent Form. Throughout the study, the recommendations of Resolution 466/2012 of the National Health Council (CNS) were followed.

### Study design, period, and location

This is an exploratory, analytical, cross-sectional study with a quantitative approach, adhering to the Strengthening the Reporting of Observational Studies in Epidemiology (STROBE) criteria^(18)^. Data were collected in September 2019 at a public school in the city of Passos, State of Minas Gerais, Brazil.

### Population and inclusion/exclusion criteria

All 1,600 enrolled students from the 6th grade of Middle School to the 3rd year of High School were invited to participate in the research, and 491 accepted. The inclusion criteria were: regular attendance, being present on the day of the questionnaire administration, age between 10 and 19 years (adolescence age range defined by the World Health Organization)^(19)^, and parental authorization for participants under 18 years old.

### Study protocol

The selection of the school for the research was based on non-probabilistic sampling using accessibility. All invited students received information about the research, and for those who expressed interest in participating, ethical requirements were followed to ensure autonomy in participation and confidentiality of the provided information.

The profiles of bullying participation (victims, bully-victims, and bullies), as well as the non-involved profile, along with the quantity and type of aggression perpetrated or experienced by the students (physical, verbal, or relational), were obtained through the application of the Peer Victimization and Aggression Scale - EVAP^(20)^. The risk of eating disorders was measured using the Eating Attitudes Test (EAT-26)^(21)^, which provided scores used to calculate group means for the total test and subscales: diet, bulimia and food preoccupation, and oral self-control.

Both instruments (EVAP and EAT-26) were administered collectively in the school during class hours. Prior to administration, researchers explained to the students how to complete the instruments, and throughout the process, they were available to clarify any doubts. Data collection in each classroom took an average of 20 minutes.

### Data analysis and statistics

The three profiles of bullying participation (victim, bully-victim, and bully) and the non-involved profile were determined through cluster analysis using the Ward hierarchical method^(22)^, which constructs clusters aiming for minimum internal variance. Analysis of variance (ANOVA) was used to identify differences in the dependent variables based on the profiles of bullying participation, followed by Bonferroni post-test to identify the groups where statistical differences were found. Spearman correlation coefficients were calculated to examine the relationships between the profiles and the risk variable for eating disorders. All analyses were performed using R 4.1.2 software, with a significance level of 5% (p < 0.05).

## RESULTS

The participants’ age (N=491) ranged from 10 to 18 years, with a mean of 14.3 years (SD=1.7 years). Regarding gender, 276 (56.2%) were female and 215 (43.8%) were male. The involvement in bullying situations was 61.1% (n=300), divided into victim-bullies (29.3%, n=144), bullies (18.1%, n=89), and victims (13.7%, n=67).

The results regarding the risk for eating disorders are presented in Table 1.

**Table 1:** Distribution of risk for eating disorders according to gender and bullying participation profile, Passos, Minas Gerais, Brazil, 2019

	Victim (n = 67)		Victim-bully (n = 144)		Bully (n = 89)		Non-involved (n = 191)	
	**n**	**%**	**n**	**%**	**n**	**%**	**n**	**%**
Risk for eating disorder								
Yes	28	41.8	38	26.4	28	31.5	32	16.8
No	39	58.2	106	73.6	61	68.5	159	83.2
Risk for eating disorder by gender								
Boys	12	42.9	9	23.7	11	39.3	9	28.1
Girls	16	57.1	29	76.3	17	60.7	23	71.9

A total of 126 participants, 25.7% of the total sample, were at risk for developing an eating disorder. The victim group had the highest percentage of risk (41.8%), while the non-involved group had the lowest percentage (16.8%), with a significant difference (p<0.001). In terms of gender, in all bullying participation profiles, girls had a higher proportion of risk for eating disorders compared to boys, as shown in Table 1.

Table 2 presents the comparisons by gender between non-involved students and involved students (victim, victim-bully, and bully) regarding the total EAT score and the subscales of risk for eating disorders.

**Table 2:** Comparison by gender between bullying-involved and non-involved students regarding total Eating Attitudes Test and subscales of risk for eating disorders, Passos, Minas Gerais, Brazil, 2019

	Victim (n = 67)		Victim-bully (n = 144)		Bully (n = 89)		Non-involved (n = 191)		Anova	
	**Mean**	**SD**	**Mean**	**SD**	**Mean**	**SD**	**Mean**	**SD**	**F**	***p* value**
Total EAT										
Boys	20.4	13.8	12.5	9.0	13.6	8.1	10.7	8.2	7.6	<0.001
Girls	19.3	9.6	17.4	9.6	17.6	9.6	13.8	9.7	4.3	0.005
Diet										
Boys	9.5	8.2	6.2	6.0	6.7	6.2	5.1	5.0	3.8	0.011
Girls	9.3	6.6	9.1	6.8	8.7	7.1	7.0	6.3	2.1	0.101
Oral control										
Boys	6.7	4.8	4.3	3.6	4.4	3.1	4.0	3.5	3.8	0.010
Girls	5.9	4.5	4.7	3.5	5.7	3.7	4.3	3.5	2.5	0.057
Bulimia and food preoccupation										
Boys	4.2	3.3	2.0	2.1	2.4	2.2	1.6	1.9	9.6	<0.001
Girls	4.1	3.0	3.6	3.0	3.3	2.8	2.5	2.7	4.1	0.007

*SD - standard deviation.*

In Table 2, the analysis of variance revealed significant differences in the mean EAT scores for boys (F=7.6, p<0.001) and girls (F=4.3, p=0.005). Bonferroni post-tests indicated that the significant difference for boys occurred between the victim group (with a higher mean) and the non-involved groups (p<0.001), victim-bullies (p=0.002), and bullies (p=0.015). For girls, there was a significant difference only between the victim group and the non-involved group (p=0.014).

Regarding the Diet subscale, there was a significant difference in the mean scores for boys (F=3.8, p=0.011), but only between victims (with a higher mean) and non-involved students (p=0.006). In the Oral Self-Control subscale, only boys’ groups showed significant differences (F=3.5, p=0.010). The differences were found between victim boys (with a higher mean) and non-involved students (p=0.003) and victim-bullies (p=0.03). Significant differences were identified in the Bulimia and Food Preoccupation subscales for boys (F=9.6, p<0.001) and girls (F=4.1, p=0.007). For boys, the significant differences occurred between the victim group (with a higher mean) and the non-involved groups (p<0.001), victim-bullies (p<0.001), and bullies (p=0.006). The only difference found for girls was between the victim group and the non-involved group (p=0.014) (Table 2).

Table 3 presents the correlations between types of victimization and aggression and the variables related to the risk for eating disorders.

**Table 3:** Correlations between variables of risk for eating disorders and types of aggression and victimization, Passos, Minas Gerais, Brazil, 2019

	Physical victimization	Verbal victimization	Relational victimization	Physical aggression	Verbal aggression	Relational aggression
Total EAT						
Boys	0.275^**^	0.230^**^	0.300^**^	0.233^**^	0.089	0.211^**^
Girls	0.141^*^	0.227^**^	0.146^*^	0.024	0.103	0.101
Diet						
Boys	0.225^**^	0.146^*^	0.274^**^	0.198^**^	0.023	0.182^**^
Girls	0.155^**^	0.136^*^	0.023	0.058	0.065	0.073
Oral control						
Boys	0.217^**^	0.165^*^	0.173^*^	0.174^*^	0.030	0.103
Girls	0.042	0.156^**^	0.172^**^	0.090	0.088	0.076
Bulimia and food preoccupation						
Boys	0.184^**^	0.276^**^	0.223^**^	0.149^*^	0.174^*^	0.194^**^
Girls	0.120^*^	0.207^**^	0.181^**^	0.073	0.040	0.073

* ^*^p<0.001;

* p<0.05.

In Table 3, the Spearman correlation coefficient indicated that, for boys, physical victimization, verbal victimization, and relational victimization were significantly correlated with all variables related to eating disorders. Physical aggression showed a significant correlation with all subscale variables of EAT-26 (eating disorder), while verbal aggression correlated significantly only with bulimia and food preoccupation. Relational aggression correlated with total EAT, diet, bulimia, and food preoccupation. In boys, the correlations were positive, indicating that the higher the level of victimization or aggression, the higher the level of eating disorders, and vice versa.

In girls, significant correlations occurred in victimization and were also positive. All three types of victimization were significantly correlated with total EAT and bulimia and food preoccupation. Additionally, in girls, physical victimization and verbal victimization showed significant correlations with diet. Verbal victimization and relational victimization were significantly correlated with oral self-control. No significant correlations were found in girls for any of the variables related to aggression and the analyzed dimensions of eating disorders.

## DISCUSSION

Our study indicates that bullying victims are at a higher risk of developing eating disorders. Similar results have been found in studies conducted in Colombia^(23)^, Chile^(24)^, Spain^(24)^, and the United States^(17)^. One possible explanation is that victims are often targeted by aggressors because they are perceived as overweight or not fitting societal beauty standards. Many instances of bullying occur due to a lack of understanding and intolerance towards differences, particularly regarding body appearance^(25)^. This may be influenced by media and societal standards that promote a thin body ideal for girls and a muscular definition for boys^(26)^.

In terms of gender, both girls and boys who are victims showed a significant risk for eating disorders. This finding, in addition to confirming the hypothesis that aggression occurs due to the weight or body appearance of the victims^(26)^, may be related to the psychological effects of victimization. Victims may develop negative body perceptions and, as a result, seek ways to alter their appearance^(17)^, such as food preoccupation, dieting, or nutritional supplementation, adopting compensatory behaviors like vomiting and laxative use, which make them more vulnerable to eating disorders.

The results regarding victims are consistent with previous studies that also did not find gender differences^(15,17,27)^. However, a different finding in this study was that male aggressors also showed a risk for eating disorders, contradicting the expectation that they would be less affected by the negative consequences of the violence they perpetrate. A study conducted in Colombia supported these results, identifying significant associations between the aggressor role and eating disorders^(23)^. Perhaps guilt and remorse for engaging in aggression against their peers lead aggressors to develop disordered eating behaviors as a way to cope with these ambivalent feelings, or they may have a heightened concern about their physical characteristics to avoid receiving back the aggression they inflict on the victims^(28)^. These results indicate that aggressors also need care to prevent eating disorders from becoming a clinical problem.

Regarding diet, only male victims, compared to victim-bullies, bullies, and non-involved students, showed significantly higher means, indicating avoidance of high-energy foods and intense concerns about physical shape. It is possible that they are seeking an “ideal body” that aligns more with their peer group’s expectations, which can result in greater social acceptance and fewer experienced aggressions^(28)^. Body-related criticisms within peer contexts, such as in bullying situations, can intensify concerns about body weight and lead to the adoption of restrictive diets. Therefore, eating disorders in males may be related to the need to feel in control, even if it is only over their weight. In the case of males, diets may not solely aim for weight loss but, in certain cases, for weight gain or muscle mass increase since in Western culture, the muscular male body is considered ideal^(29)^.

Male victims also exhibited significantly greater food control, indicating a heightened sense of environmental and social pressures related to food intake. This may be related to the diet result, which, in turn, may stem from the greater violence suffered concerning weight and physical appearance. For boys, all types of bullying victimization (physical, verbal, and psychological) were correlated with oral self-control, whereas for girls, there was no correlation with physical victimization. This may suggest that boys, in addition to being targets of body-related derogation in the bullying context, also experience physical aggression such as hitting, punching, kicking, among others^(4)^. As the results indicate, they may more actively seek to alter their body weight through diets and, consequently, feel more pressured by their social group, family, or friends to consume food.

Both female and male victims showed significant risk for binge-eating episodes followed by compensatory behaviors to avoid weight gain. In Copeland et al.’s study^(17)^, victims were also more vulnerable to symptoms of bulimia.

Overall, the results indicate that, in the investigated sample, male victims of bullying are more vulnerable to the development of eating disorders. This is concerning since adolescence is considered a high-risk period for the development of psychological disorders due to various ongoing transformations: physical, psychological, and social, which directly influence body image acceptance^(30)^. Therefore, experiencing bullying can exacerbate the challenges faced by some adolescents, making them even more vulnerable to eating disorders. This situation is particularly worrisome for the boys in the study, as although men are now encouraged to seek treatment for eating disorders, women are still more likely to seek help^(31)^.

In this regard, it is important to highlight that men’s health is a field still under development, and the issue of eating disorders in this population is generally underdiagnosed, undertreated, and poorly understood. Furthermore, the aggression suffered related to body size or shape may lead adolescents to seek quick solutions that can be detrimental to their health, such as inappropriate diets^(32)^.

### Study limitations

The sample size used limits the generalizability of our results. The cross-sectional nature of the study does not allow for establishing causal relationships, although the identified associations have significant scientific and clinical relevance. Another limitation is that the data were collected through self-report instruments, which may introduce memory bias or result in lower participant engagement when answering the questions, particularly for student aggressors who may have concerns about being recognized in some way, even if they are not identified in the questionnaires. However, these instruments are validated for the Brazilian population, and we have no reason to consider their low community relevance.

### Contributions to the Nursing, Health, or Public Policy field

The study addresses a topic included in national and international proposals and initiatives promoting a culture of peace and health in schools, such as the Regional Initiative Schools Promoting Health in the Americas, Health-Promoting Schools, and the Health in School Program^(32)^. In this context, promoting health in schools also ensures the individual and collective empowerment and emancipation of students, particularly in contributing to breaking prejudice, discrimination, and cycles of systematic violence like bullying.

The National Policy for Health Promotion (PNPS) also prioritizes the promotion of a culture of peace and good coexistence, which involves, among other aspects, promoting respect for diversity and reducing violence^(33)^. However, these topics are still underexplored in the training and professional practice of nurses^(34)^. Studies indicate that the coordinated and intersectoral action of Primary Health Care (PHC) professionals in the area of violence against children and adolescents remains a challenge^(34-35)^.

Thus, improvements in the quality of students’ social interactions can be developed by nurses and other health professionals through school health promotion activities that encourage good coexistence and the establishment of a culture of non-violence. This not only prevents and breaks cycles of violence among students but also prevents other negative outcomes of school bullying, such as the development of eating disorders.

## CONCLUSIONS

The risk of developing eating disorders was higher for the victim profile, both for males and females. Associations between boys and aggression were also observed. The results indicate that victims, especially boys, are more vulnerable to the negative consequences of bullying regarding the risk of eating disorders.

Considering that eating disorders typically emerge during adolescence, a period when bullying is more prevalent, identifying factors associated with bullying can improve understanding of this type of violence and provide a basis for the development of anti-bullying interventions and eating disorder prevention. These interventions should be interdisciplinary, promoting non-violence and health, with proposals and approaches based on the integration of different sectors and equity, as bullying affects students in various ways, whether they are victims, aggressors, victim-bullies, or witnesses. Simultaneously, providing care for cases of bullying and eating disorders requires defining target groups, and this study can assist Primary Care teams in this process.

## Supplementary Material

0034-7167-reben-76-05-e20220643-sup01

## Data Availability

https://doi.org/10.48331/scielodata.AMEZXY
