# Supplementary material for: Associations between bullying and risk for eating disorders in adolescents
Source: Rev Bras Enferm. 2023 Nov 27;76(5):e20220643. doi: 10.1590/0034-7167-2022-0643 (PMC10680392; doi:10.1590/0034-7167-2022-0643)
Supplement: 0034-7167-reben-76-05-e20220643-sup01 [file 0034-7167-reben-76-05-e20220643-sup01.pdf]

Sujeito	Perfil de participação no bullying	Ano escolar	Idade	Sexo	NSE	Reprovação
1	1	2	12	2	1	1
2	1	2	13	1	3	1
3	4	2	12	1	3	1
4	1	2	12	2	3	1
5	1	2	12	2	1	1
6	4	2	12	2	2	1
7	1	2	12	1	2	1
8	4	2	12	1	1	1
9	3	2	12	2	3	1
10	4	2	12	2	1	1
11	4	2	12	2	1	1
12	2	2	12	2	1	1
13	1	2	12	2	1	1
14	3	2	12	2	2	1
15	2	2	12	2	3	1
16	1	2	12	1	5	1
17	4	2	12	1	1	1
18	4	2	12	2	3	1
19	3	2	12	2	2	1
20	4	2	12	1	1	1
21	4	2	12	2	1	1
22	2	2	12	2	2	1
23	1	2	12	1	1	1
24	1	2	12	1	2	1
25	1	2	12	2	2	1
26	2	2	12	2	1	1
27	4	2	13	1	1	1
28	2	2	12	1	2	1
29	4	2	12	2	1	1
30	3	2	12	2	3	1
31	1	2	12	1	3	1
32	1	2	12	2	4	1
33	4	2	12	2	4	1
34	4	2	12	2	5	1
35	1	2	12	2	5	1
36	1	2	12	2	1	1
37	4	2	12	2	2	1
38	2	2	13	2	2	2
39	2	2	12	1	5	1
40	1	2	12	1	4	1
41	4	2	12	1	5	1
42	1	2	13	2	5	1
43	4	2	12	2	4	1
44	1	2	13	2	2	1
45	4	2	12	2	4	1
46	1	2	12	1	5	1

47	1	2	13	2	5	1
48	1	2	12	2	4	1
49	1	2	12	2	4	1
50	2	2	12	1	3	1
51	3	2	12	1	3	1
52	1	2	12	2	3	1
53	2	2	12	2	1	1
54	4	2	12	2	1	1
55	4	2	12	2	1	1
56	2	2	12	2	4	1
57	3	2	13	1	4	1
58	4	2	12	1	3	1
59	1	2	12	1	3	1
60	4	2	12	1	3	1
61	3	2	12	1	3	1
62	2	2	12	1	4	1
63	2	2	12	1	2	1
64	2	2	12	1	2	1
65	4	2	12	2	1	1
66	2	2	12	1	4	1
67	4	2	12	2	3	1
68	2	2	12	2	3	1
69	3	2	12	1	3	1
70	3	2	12	1	3	1
71	2	2	13	2	1	1
72	3	2	12	1	2	1
73	2	2	12	2	3	1
74	4	2	12	1	3	1
75	1	2	13	1	5	1
76	1	2	12	2	3	1
77	1	2	12	2	1	1
78	1	2	12	2	1	1
79	1	2	12	1	2	1
80	1	2	12	1	3	1
81	1	2	12	2	1	1
82	1	2	12	2	4	1
83	1	2	12	2	5	1
84	1	2	12	2	5	1
85	1	2	12	2	5	1
86	4	2	12	1	1	1
87	1	2	12	1	3	1
88	3	2	13	1	4	1
89	3	2	12	1	1	1
90	1	2	12	1	3	1
91	1	2	12	2	3	1
92	4	2	12	2	1	1
93	1	2	15	2	3	1

94	4	2	12	2	4	1
95	1	2	12	2	1	1
96	1	2	12	2	2	1
97	1	2	12	1	4	1
98	2	2	12	1	5	1
99	1	2	12	1	4	1
100	1	2	12	2	5	1
101	1	2	12	2	5	1
102	1	2	12	2	1	1
103	4	2	13	1	3	1
104	1	2	12	2	3	1
105	4	3	13	1	1	1
106	4	3	14	2	3	1
107	4	3	13	2	2	1
108	1	3	13	2	1	1
109	3	3	13	2	3	1
110	3	3	13	2	3	1
111	2	3	13	1	3	1
112	1	3	13	2	4	1
113	4	3	13	2	1	1
114	1	3	13	1	2	1
115	1	3	13	1	2	1
116	4	3	14	2	5	1
117	4	3	13	1	2	1
118	2	3	13	2	3	1
119	1	3	14	1	1	1
120	1	3	15	1	4	1
121	3	3	14	2	4	1
122	1	3	13	1	4	1
123	2	3	13	1	4	1
124	1	3	13	1	3	1
125	4	3	13	1	5	1
126	1	3	13	2	5	1
127	1	3	13	2	3	1
128	1	3	13	2	4	1
129	3	3	13	1	3	1
130	3	3	13	2	3	1
131	4	3	13	2	1	1
132	4	3	13	1	3	1
133	4	3	13	2	2	1
134	2	3	14	2	5	1
135	3	3	14	2	5	1
136	2	3	14	2	4	1
137	1	3	13	2	5	1
138	3	3	12	2	2	1
139	1	3	12	1	2	1
140	1	3	12	1	2	1

141	1	3	13	1	1	1
142	1	3	13	2	2	1
143	4	3	14	2	2	1
144	1	3	14	1	2	2
145	1	3	12	2	1	1
146	3	3	12	1	2	1
147	1	3	13	1	1	1
148	2	3	13	2	1	1
149	3	3	13	2	1	1
150	3	3	13	2	2	1
151	4	3	13	2	3	1
152	1	3	13	1	2	1
153	4	3	14	2	2	1
154	3	3	13	2	1	1
155	4	3	13	2	4	1
156	3	3	13	1	1	1
157	3	3	13	2	2	1
158	4	3	13	1	1	1
159	2	3	13	1	2	1
160	3	3	13	2	1	1
161	1	3	13	2	1	1
162	3	3	13	1	3	1
163	1	3	12	2	3	1
164	2	3	13	2	3	1
165	1	3	13	2	1	1
166	4	3	13	2	2	1
167	1	3	13	2	3	1
168	1	3	13	2	3	1
169	3	3	13	2	5	1
170	3	3	13	1	2	1
171	1	4	14	2	3	1
172	3	4	14	1	5	1
173	3	4	14	2	4	1
174	1	4	14	1	2	1
175	4	4	14	1	3	1
176	2	4	14	1	1	1
177	4	4	14	1	1	1
178	4	4	14	1	3	1
179	4	4	14	1	2	1
180	3	4	14	1	3	1
181	4	4	14	2	1	1
182	1	4	14	2	1	1
183	3	4	15	2	1	1
184	3	4	14	1	4	1
185	1	4	14	1	3	1
186	2	4	15	2	4	2
187	4	4	14	1	1	1

188	3	4	14	1	5	1
189	2	4	14	1	1	1
190	2	4	13	1	3	1
191	4	4	13	1	1	1
192	1	4	14	2	5	1
193	1	4	14	2	5	1
194	1	4	14	2	2	1
195	4	4	14	2	3	1
196	4	4	14	2	1	1
197	1	4	13	1	1	1
198	4	4	14	1	4	1
199	3	4	14	1	4	1
200	4	4	14	2	1	1
201	4	4	14	2	4	1
202	1	4	14	2	2	1
203	1	4	14	2	3	1
204	4	4	14	1	4	1
205	1	4	13	2	3	1
206	2	4	14	2	2	1
207	4	4	14	1	2	1
208	4	4	13	1	1	1
209	1	4	14	2	4	1
210	4	4	14	1	1	1
211	2	4	14	2	1	1
212	4	4	14	1	1	1
213	2	4	14	2	3	1
214	3	4	14	1	2	1
215	4	4	14	2	1	1
216	1	4	14	1	1	1
217	4	4	16	1	1	1
218	1	4	14	2	5	2
219	4	4	14	2	2	1
220	1	4	15	1	3	2
221	4	4	14	1	4	1
222	1	4	14	1	1	1
223	1	4	14	2	3	1
224	1	4	14	1	4	1
225	1	4	14	1	2	1
226	3	4	14	1	4	1
227	2	4	16	2	2	2
228	1	4	15	1	1	2
229	1	4	14	2	3	1
230	4	4	14	2	3	1
231	2	4	14	1	4	1
232	1	4	14	2	3	1
233	3	4	13	2	3	1
234	2	4	15	2	2	1

235	2	4	14	2	5	2
236	4	4	14	1	2	1
237	4	4	14	1	2	1
238	1	4	14	2	3	1
239	3	4	14	2	2	1
240	1	4	14	2	1	1
241	4	4	14	2	2	1
242	4	4	14	2	1	1
243	4	4	14	2	1	1
244	3	4	14	2	3	1
245	4	4	14	2	3	1
246	4	4	14	2	2	1
247	4	4	14	2	2	1
248	1	4	14	2	1	1
249	4	4	14	1	1	1
250	1	4	14	2	3	1
251	2	4	16	1	4	2
252	4	4	14	1	5	1
253	4	4	14	2	1	1
254	3	4	14	1	1	1
255	1	4	14	1	1	1
256	3	4	14	1	4	1
257	1	4	14	1	5	1
258	4	4	14	2	2	1
259	1	4	14	2	3	1
260	1	4	14	1	1	1
261	1	4	13	1	1	1
262	2	4	14	1	3	1
263	4	4	14	2	5	1
264	1	4	14	1	1	1
265	1	4	13	2	1	1
266	4	5	15	2	4	1
267	1	5	15	2	5	1
268	1	5	15	2	1	2
269	4	5	15	2	5	1
270	1	5	15	2	3	1
271	1	5	15	2	3	1
272	1	5	15	2	2	1
273	2	5	15	1	1	1
274	1	5	15	1	1	1
275	4	5	15	1	3	1
276	1	5	15	1	1	1
277	4	5	15	1	3	1
278	3	5	15	1	1	4
279	4	5	15	2	4	1
280	2	5	16	2	3	1
281	4	5	15	2	3	1

282	1	5	16	2	1	1
283	4	5	15	1	2	1
284	3	5	14	1	2	1
285	4	5	14	2	2	2
286	1	5	15	2	2	1
287	4	5	15	2	2	1
288	1	5	14	1	2	1
289	3	5	15	1	3	1
290	1	5	15	1	4	1
291	3	5	15	2	3	1
292	1	5	15	2	3	1
293	2	5	15	1	1	1
294	4	5	15	2	2	1
295	1	5	14	2	1	1
296	1	5	15	1	4	1
297	3	5	15	2	4	1
298	4	5	15	1	2	1
299	4	5	15	2	4	1
300	1	5	15	2	3	1
301	4	5	15	1	1	1
302	3	5	15	1	3	1
303	1	5	15	1	4	1
304	1	5	15	1	1	1
305	3	5	15	1	3	1
306	4	5	15	2	3	1
307	4	5	15	1	3	1
308	1	5	15	2	3	1
309	4	5	14	2	1	1
310	4	5	14	2	1	1
311	2	5	15	2	1	1
312	1	5	15	2	2	1
313	3	5	14	2	2	1
314	4	5	15	2	2	1
315	4	5	15	2	1	1
316	1	5	15	2	1	1
317	1	5	15	2	4	1
318	3	5	15	1	2	1
319	4	5	15	1	3	1
320	4	5	15	1	2	1
321	3	5	15	1	4	1
322	2	5	15	1	3	1
323	2	5	14	2	3	1
324	4	5	15	1	3	1
325	1	5	15	1	1	1
326	1	5	15	1	2	2
327	2	5	15	1	3	1
328	2	5	15	1	3	1

329	1	5	15	1	2	1
330	4	5	15	2	5	1
331	2	5	15	2	2	1
332	2	5	14	2	3	1
333	4	5	14	1	4	1
334	3	5	14	2	3	1
335	1	5	15	1	2	1
336	1	5	15	1	4	1
337	4	5	15	1	2	1
338	3	5	15	1	2	1
339	1	5	15	1	2	1
340	3	5	15	1	4	1
341	3	5	15	1	2	1
342	4	5	15	1	4	1
343	4	5	15	2	5	1
344	3	5	15	2	2	1
345	2	5	16	2	3	2
346	2	5	15	2	4	1
347	4	5	16	1	1	1
348	3	5	15	2	3	1
349	4	5	15	2	5	1
350	4	5	15	1	3	1
351	4	5	15	1	3	2
352	4	5	15	2	3	1
353	3	5	16	1	2	1
354	3	6	16	2	3	1
355	2	6	16	1	2	1
356	4	6	16	2	3	1
357	3	6	16	1	5	1
358	1	6	16	2	2	1
359	4	6	17	2	1	1
360	4	6	16	2	1	1
361	1	6	15	2	1	1
362	1	6	16	2	1	1
363	1	6	16	2	2	1
364	1	6	16	2	4	1
365	1	6	16	1	1	1
366	2	6	16	1	4	1
367	4	6	16	2	2	1
368	1	6	16	1	4	1
369	4	6	16	1	5	1
370	1	6	16	2	1	1
371	3	6	16	2	3	1
372	1	6	16	1	3	1
373	3	6	16	2	1	1
374	4	6	16	1	1	1
375	3	6	16	2	5	1

376	1	6	16	1	1	1
377	3	6	16	1	2	1
378	2	6	16	2	3	1
379	3	6	15	1	4	1
380	2	6	16	2	1	1
381	1	6	16	2	1	1
382	1	6	17	1	1	1
383	3	6	16	1	2	1
384	4	6	15	2	3	1
385	3	6	15	1	3	1
386	3	6	15	2	3	1
387	4	6	16	2	2	1
388	1	6	16	2	4	1
389	1	6	16	2	3	1
390	4	6	16	2	4	1
391	3	6	16	2	5	1
392	4	6	14	1	1	1
393	1	6	16	1	3	1
394	4	6	16	2	4	1
395	1	6	16	2	1	1
396	3	6	17	1	2	1
397	4	6	15	1	2	1
398	4	6	15	1	3	1
399	3	6	16	1	1	1
400	4	6	16	1	1	1
401	1	6	16	1	1	1
402	4	6	16	2	3	1
403	4	6	16	1	1	1
404	1	6	16	2	3	1
405	2	6	15	1	2	1
406	2	6	17	2	3	2
407	4	6	16	2	4	1
408	4	6	16	2	1	1
409	2	6	16	2	3	1
410	3	6	16	2	5	1
411	2	6	16	1	1	1
412	1	6	16	2	4	1
413	4	6	16	2	2	1
414	1	6	16	2	3	1
415	1	6	16	2	2	1
416	1	6	16	2	1	1
417	3	6	16	1	3	1
418	1	6	16	2	4	1
419	4	6	17	2	5	1
420	1	6	16	2	4	1
421	1	6	16	1	1	1
422	1	6	16	1	1	1

423	2	6	16	2	1	1
424	3	6	15	2	3	1
425	1	6	16	1	4	1
426	2	6	16	2	1	1
427	4	6	16	2	2	1
428	1	7	17	1	1	1
429	2	7	17	2	3	1
430	3	7	18	2	3	2
431	1	7	17	1	1	1
432	4	7	19	2	4	1
433	1	7	17	2	1	1
434	3	7	17	1	2	1
435	1	7	17	2	3	1
436	3	7	17	2	2	1
437	3	7	17	1	2	1
438	4	7	17	1	1	1
439	1	7	17	1	4	1
440	4	7	17	1	1	1
441	1	7	17	1	3	1
442	3	7	17	2	1	1
443	2	7	17	2	2	1
444	4	7	17	2	1	1
445	4	7	17	2	3	1
446	1	7	17	2	3	1
447	3	7	17	1	2	1
448	1	7	17	2	2	1
449	4	7	17	1	1	1
450	3	7	17	2	3	1
451	1	7	17	1	3	1
452	4	7	17	2	2	1
453	3	7	17	1	2	1
454	4	7	17	2	2	1
455	3	7	17	2	3	1
456	4	7	17	2	2	1
457	2	7	18	2	2	1
458	2	7	16	2	2	1
459	1	7	17	1	2	1
460	4	7	17	2	2	1
461	4	7	17	2	2	1
462	1	7	17	2	2	1
463	1	7	17	2	2	1
464	3	7	17	2	5	1
465	1	7	17	1	1	1
466	1	7	17	2	3	1
467	1	7	17	1	2	1
468	1	7	17	2	4	1
469	2	7	18	1	3	2

470	3	7	17	2	3	1
471	3	7	17	2	4	1
472	3	7	16	1	1	1
473	2	7	17	1	3	1
474	1	7	17	1	2	1
475	1	7	17	2	2	1
476	1	7	17	1	3	1
477	1	7	17	2	3	1
478	1	7	17	2	4	1
479	4	7	17	1	3	1
480	1	7	17	2	4	1
481	1	7	17	2	2	1
482	1	7	16	1	2	1
483	1	7	16	2	2	1
484	1	7	17	2	2	1
485	1	7	17	1	3	1
486	1	7	17	2	2	1
487	1	7	17	1	3	1
488	1	7	17	2	3	1
489	1	7	17	1	3	1
490	1	7	17	1	1	1
491	1	7	17	1	1	1

D-E
C2
C1
B2
B1
A

Cor	Agressão total	Vitimização Total	Agressão física	Agressão verbal	Agressão relacional
3	17	11	5	8	4
1	15	11	5	5	5
2	11	15	4	4	3
3	10	8	3	4	3
3	10	8	3	4	3
1	10	17	3	4	3
3	15	8	3	8	4
3	11	17	4	4	3
2	20	17	5	9	6
2	14	20	4	7	3
2	17	13	3	8	6
1	14	37	3	8	3
1	10	8	3	4	3
2	21	16	4	14	3
3	20	25	5	9	6
1	13	11	3	7	3
1	18	13	3	9	6
3	12	18	3	6	3
3	19	20	3	12	4
5	18	17	6	7	5
3	13	16	3	5	5
3	37	32	9	18	10
3	14	8	3	5	6
3	14	8	3	7	4
3	11	10	3	5	3
1	15	25	3	9	3
2	15	15	7	5	3
1	18	23	5	9	4
1	12	14	4	5	3
3	21	19	4	13	4
3	18	9	4	10	4
3	10	10	3	4	3
1	16	14	5	6	5
1	10	16	3	4	3
3	10	10	3	4	3
5	15	8	5	7	3
3	16	14	7	6	3
3	33	38	9	15	9
1	14	25	4	7	3
3	11	12	3	5	3
1	10	15	3	4	3
3	10	11	3	4	3
3	16	19	6	7	3
3	10	11	3	4	3
3	16	16	4	7	5
3	10	8	3	4	3

3	19	10	4	11	4
1	13	12	3	7	3
3	10	8	3	4	3
2	22	24	5	12	5
3	27	19	7	12	8
1	10	8	3	4	3
3	23	26	5	12	6
3	11	14	3	6	2
3	16	16	5	8	3
1	22	26	5	14	3
1	34	20	7	20	7
3	22	15	9	6	7
1	17	11	3	10	4
3	16	14	7	6	3
3	22	20	3	12	7
3	31	29	4	15	12
2	18	25	7	8	3
5	29	30	6	17	6
3	10	16	3	4	3
2	28	30	7	10	11
3	13	20	3	7	3
3	23	22	6	10	7
3	29	19	5	13	11
1	26	14	7	12	7
1	20	24	6	10	4
3	24	14	3	14	7
3	15	26	5	6	4
1	16	20	5	8	3
1	10	8	3	4	3
3	10	8	3	4	3
1	10	8	3	4	3
2	18	10	5	8	5
1	10	8	3	4	3
1	12	11	3	6	3
3	12	8	3	6	3
1	18	8	7	8	3
1	10	8	3	4	3
1	14	9	4	7	3
3	12	8	3	6	3
1	11	15	2	6	3
1	14	10	4	7	3
3	30	19	9	13	8
1	20	11	4	12	4
1	10	8	3	4	3
3	13	10	3	7	3
3	13	13	4	6	3
1	10	8	3	4	3

1	22	19	9	8	5
1	10	12	3	4	3
1	10	10	3	4	3
3	14	10	4	7	3
3	28	31	7	13	8
3	11	8	3	4	4
1	14	12	5	5	4
1	13	11	5	5	3
1	12	8	4	5	3
1	13	14	3	7	3
3	10	8	3	4	3
3	18	19	3	8	7
3	10	14	3	4	3
5	11	20	3	5	3
3	11	8	3	5	3
3	20	16	5	12	3
2	22	14	3	14	5
3	26	25	8	11	7
3	12	12	5	4	3
3	12	16	3	6	3
3	11	8	3	4	4
3	10	8	3	4	3
1	10	17	3	4	3
3	15	18	3	8	4
3	14	35	7	4	3
5	10	8	3	4	3
2	18	8	7	8	3
5	38	22	9	20	9
3	10	9	3	4	3
3	21	24	5	11	5
5	10	8	3	4	3
3	20	17	4	8	8
3	13	9	5	5	3
1	21	8	4	10	7
1	13	8	3	7	3
3	37	17	10	20	7
3	39	20	10	19	10
3	15	21	3	7	5
3	22	13	9	8	5
1	10	16	3	4	3
3	30	37	11	12	7
3	25	18	3	15	7
3	23	24	7	10	6
2	19	9	4	11	4
3	31	17	7	15	9
3	10	12	3	4	3
5	15	11	3	7	5

3	13	9	3	7	3
3	12	8	3	6	3
1	15	18	5	6	4
1	11	12	3	5	3
2	12	8	3	6	3
3	25	21	5	13	7
3	13	8	4	5	4
1	16	22	4	9	3
1	18	19	4	10	4
2	19	12	4	12	3
3	12	17	4	5	3
3	13	8	3	7	3
3	12	19	3	6	3
1	18	12	4	11	3
1	14	16	3	7	4
3	22	20	5	12	5
1	20	14	3	10	7
1	12	15	3	6	3
1	21	22	3	11	7
1	35	19	6	19	10
4	13	11	4	6	3
3	21	20	4	11	6
1	10	8	3	4	3
3	19	24	7	9	3
3	11	11	4	4	3
3	15	13	5	6	4
3	12	8	3	6	3
3	13	11	3	6	4
1	20	17	6	11	3
3	25	15	7	11	7
3	10	10	3	4	3
3	21	14	6	12	3
3	30	8	6	20	4
1	12	11	4	5	3
3	17	15	4	10	3
1	34	25	13	12	9
1	14	15	3	5	6
2	16	13	3	10	3
1	17	13	4	10	3
x	24	14	8	11	5
2	14	13	4	6	4
3	13	8	3	7	3
1	18	18	3	12	3
3	23	18	5	12	6
3	10	8	3	4	3
2	12	23	3	6	3
1	10	13	3	4	3

3	19	20	6	10	3
3	13	27	5	4	4
2	29	28	6	14	9
3	17	15	5	7	5
3	11	8	4	4	3
3	10	12	3	4	3
3	13	11	3	7	3
3	12	21	3	6	3
3	17	13	3	7	7
3	18	11	5	9	4
2	16	17	6	7	3
2	20	17	4	11	5
1	11	18	3	6	2
1	12	18	4	5	3
3	13	10	4	6	3
3	10	11	3	4	3
3	14	15	4	6	4
1	10	12	3	4	3
3	19	31	4	10	5
1	17	12	3	10	4
1	15	14	3	9	3
3	19	8	5	10	4
3	16	14	7	6	3
3	16	22	5	8	3
3	11	13	4	4	3
2	25	27	5	15	5
3	33	21	11	13	9
1	15	18	7	5	3
2	12	10	3	6	3
2	13	15	3	5	5
1	10	8	3	4	3
1	19	21	7	8	4
2	17	8	4	8	5
3	13	14	5	5	3
3	16	10	6	7	3
1	10	8	3	4	3
3	17	12	5	7	5
3	10	8	3	4	3
1	24	14	7	12	5
1	20	31	3	13	4
1	10	8	3	4	3
1	10	11	3	4	3
2	12	16	3	7	2
3	25	25	8	10	7
1	11	10	3	5	3
3	21	17	5	12	4
3	13	23	4	6	3

3	17	25	5	8	4
1	13	14	3	7	3
4	16	16	3	10	3
2	10	8	3	4	3
3	20	18	5	10	5
3	10	12	3	4	3
3	13	19	3	7	3
1	13	20	4	6	3
3	12	18	4	5	3
3	21	20	6	12	3
1	13	13	3	7	3
3	12	16	5	4	3
4	14	15	4	6	4
3	14	10	4	7	3
3	14	19	4	7	3
1	10	8	3	4	3
1	28	28	9	9	10
2	13	16	3	7	3
3	10	14	3	4	3
3	20	17	6	9	5
3	12	8	3	5	4
3	21	11	5	13	3
3	10	8	3	4	3
3	15	14	3	7	5
3	11	8	3	5	3
1	12	8	3	6	3
3	14	10	2	9	3
4	11	23	3	4	4
3	11	14	3	5	3
1	10	10	3	4	3
1	13	12	3	6	4
3	14	15	3	8	3
3	10	8	3	4	3
2	10	8	3	4	3
1	12	14	3	6	3
1	10	8	3	4	3
1	18	8	3	12	3
1	12	11	4	4	4
3	16	22	3	10	3
2	20	10	5	12	3
3	15	14	3	9	3
1	14	8	3	8	3
3	19	16	6	8	5
3	21	14	7	10	4
1	12	19	3	6	3
1	12	27	5	4	3
3	19	17	5	9	5

3	12	10	4	5	3
3	13	15	3	7	3
3	29	19	5	13	11
3	15	13	3	9	3
3	11	8	3	5	3
2	15	15	4	7	4
1	16	10	3	8	5
3	20	14	3	12	5
3	14	8	3	8	3
3	29	15	8	12	9
3	12	8	5	4	3
1	14	25	4	7	3
2	21	16	8	8	5
1	14	10	5	6	3
1	11	10	3	5	3
3	27	18	6	10	11
1	18	13	4	10	4
1	18	17	6	8	4
3	13	9	3	7	3
3	15	13	4	7	4
1	19	12	3	12	4
3	12	10	4	5	3
1	12	10	3	5	4
3	20	15	4	10	6
3	10	16	3	4	3
3	18	14	5	9	4
3	12	12	5	4	3
3	19	14	6	9	4
1	11	14	3	4	4
1	22	21	7	8	7
3	10	12	3	4	3
3	20	18	5	11	4
3	17	14	7	7	3
1	15	13	6	6	3
3	11	10	3	5	3
3	15	8	3	7	5
1	23	17	7	11	5
1	17	13	3	9	5
3	13	17	4	6	3
1	18	14	3	12	3
1	14	23	5	5	4
3	20	25	6	9	5
3	12	13	4	5	3
3	12	12	3	6	3
1	12	8	3	6	3
3	18	22	3	10	5
1	16	32	6	4	6

1	14	8	5	6	3
3	11	13	3	5	3
1	15	23	5	7	3
3	14	24	3	6	5
3	14	13	3	7	4
3	22	14	4	13	5
1	15	11	4	6	5
1	11	12	3	5	3
3	12	16	3	6	3
2	23	20	5	12	6
2	11	12	3	5	3
3	22	12	5	10	7
3	19	12	4	12	3
3	16	14	4	9	3
1	13	17	4	6	3
1	19	12	3	12	4
1	10	26	3	4	3
3	50	40	15	20	15
2	14	13	4	7	3
1	22	14	4	12	6
3	22	16	14	6	2
2	14	16	4	6	4
3	19	14	6	6	7
1	11	19	4	4	3
3	22	17	6	11	5
1	25	17	2	18	5
1	20	28	5	11	4
1	16	18	3	8	5
1	21	16	5	13	3
1	11	11	3	5	3
3	10	13	3	4	3
3	16	16	4	7	5
1	10	11	3	4	3
3	11	11	3	5	3
1	10	12	3	4	3
1	11	8	3	5	3
1	15	8	3	9	3
3	20	25	7	9	4
3	13	13	4	5	4
1	13	12	3	7	3
3	19	15	5	9	5
1	13	9	4	6	3
1	17	20	3	11	3
2	12	10	3	6	3
3	17	17	3	10	4
2	15	14	3	9	3
1	18	18	3	11	4

2	12	12	6	5	1
3	25	13	5	13	7
3	20	33	5	12	3
3	21	13	4	11	6
1	21	28	3	13	5
3	10	12	3	4	3
3	12	10	3	6	3
3	23	12	6	14	3
2	14	18	3	7	4
1	36	20	8	17	11
2	24	18	5	12	7
3	16	16	4	8	4
1	16	8	3	9	4
3	15	12	5	6	4
3	19	20	6	7	6
3	21	19	6	10	5
3	12	14	3	5	4
3	22	11	7	9	6
3	17	15	6	8	3
2	12	8	3	5	4
2	24	11	7	14	3
1	14	13	3	6	5
2	18	12	4	10	4
2	22	15	5	11	6
3	10	17	3	4	3
2	15	9	3	9	3
3	14	13	4	6	4
3	20	13	7	9	4
3	10	11	3	4	3
1	15	27	3	9	3
3	19	22	6	7	6
3	17	18	5	9	3
3	14	13	5	6	3
3	17	24	3	10	4
1	21	16	5	12	4
11	10	31	3	4	3
3	10	8	3	4	3
1	20	12	8	9	3
3	13	10	3	6	4
3	14	11	4	6	4
3	21	11	7	6	8
3	21	17	4	12	5
1	10	8	3	4	3
2	14	14	5	6	3
1	12	10	3	4	5
1	13	8	3	7	3
1	22	8	7	12	3

1	19	36	7	8	4
1	18	14	3	11	4
1	10	8	3	4	3
3	16	28	5	8	3
1	13	14	3	6	4
3	11	12	3	5	3
2	12	25	3	6	3
3	20	16	3	14	3
1	15	8	3	9	3
2	11	13	4	4	3
1	10	9	3	4	3
2	20	14	5	10	5
3	14	8	7	4	3
2	23	12	3	11	9
3	20	13	5	10	5
2	14	15	3	8	3
1	14	11	4	6	4
3	16	20	3	9	4
2	17	10	3	11	3
2	17	20	4	10	3
3	16	30	7	4	5
1	13	14	4	5	4
2	17	15	4	7	6
3	12	11	3	6	3
3	22	16	5	14	3
3	15	11	5	5	5
3	12	14	3	5	4
3	18	18	4	11	3
2	10	8	3	4	3
2	13	19	4	6	3
1	22	12	5	12	5
3	12	13	4	5	3
3	20	18	3	14	3
1	14	16	5	5	4
3	16	27	3	8	5
3	17	27	4	8	5
1	10	8	3	4	3
1	12	19	4	5	3
2	13	15	3	7	3
3	10	8	3	4	3
1	10	9	3	4	3
4	22	17	5	14	3
3	10	8	3	4	3
1	12	10	3	6	3
1	10	8	3	4	3
3	14	9	5	6	3
4	22	28	3	12	7

1	25	21	7	12	6
1	18	19	3	12	3
5	20	21	3	14	3
1	10	34	3	4	3
1	12	8	3	6	3
1	12	10	3	6	3
1	18	8	6	9	3
1	10	10	3	4	3
1	10	10	3	4	3
3	10	14	3	4	3
1	10	8	3	4	3
1	15	11	3	9	3
1	13	8	3	6	4
2	12	11	3	6	3
1	10	8	3	4	3
2	10	10	3	4	3
1	12	8	3	6	3
1	16	10	3	9	4
1	10	9	3	4	3
1	11	8	3	5	3
3	10	8	3	4	3
1	10	8	3	4	3

Vitimização física	Vitimização verbal	Vitimização relacional	Bulimia	Controle oral	Dieta	EAT
2	6	3	0	4	1	5
2	7	2	5	11	14	30
3	9	3	4	3	4	11
2	4	2	6	8	3	17
2	4	2	0	2	3	5
3	9	5	1	12	5	18
2	4	2	2	0	6	8
3	11	3	1	6	6	13
2	11	4	0	0	3	3
4	12	4	1	3	6	10
3	8	2	7	4	9	20
10	20	7	0	6	0	6
2	4	2	5	3	10	18
2	10	4	7	2	16	25
6	12	7	4	4	15	23
3	6	2	2	9	15	26
2	9	2	1	1	4	6
4	10	4	5	15	9	29
2	16	2	3	6	12	21
2	11	4	1	12	10	23
4	8	4	5	4	6	15
9	14	9	1	3	10	14
2	4	2	4	8	4	16
2	4	2	4	1	4	9
3	5	2	4	7	6	17
6	16	3	4	4	19	27
3	8	4	3	4	12	19
6	13	4	6	6	6	18
3	7	4	0	1	2	3
3	14	2	1	6	4	11
2	5	2	12	15	18	45
4	4	2	0	4	7	11
2	9	3	0	6	3	9
5	9	2	2	4	2	8
4	4	2	1	4	14	19
2	4	2	0	1	4	5
5	6	3	6	6	7	19
9	20	9	3	17	6	26
7	12	6	1	5	7	13
4	6	2	0	6	4	10
5	7	3	1	2	2	5
2	6	3	6	12	14	32
5	11	3	0	2	7	9
2	6	3	8	6	13	27
4	7	5	1	2	4	7
2	4	2	0	6	4	10

2	6	2	9	0	6	15
2	7	3	9	11	22	42
2	4	2	1	0	6	7
6	15	3	7	9	5	21
6	9	4	9	9	5	23
2	4	2	0	3	11	14
6	18	2	6	5	16	27
4	8	2	0	1	5	6
2	12	2	0	2	3	5
8	16	2	3	15	7	25
6	12	2	0	7	19	26
5	4	6	3	4	11	18
2	6	3	1	3	15	19
4	8	2	5	5	13	23
6	12	2	4	6	10	20
6	18	5	12	13	9	34
5	18	2	6	9	22	37
6	16	8	6	3	18	27
3	11	2	0	2	7	9
6	20	4	12	16	24	52
6	12	2	3	4	18	25
6	13	3	0	1	5	6
4	9	6	6	5	19	30
4	8	2	3	4	6	13
5	15	4	3	8	18	29
4	8	2	0	1	3	4
6	17	3	0	2	3	5
5	12	3	1	4	2	7
2	4	2	3	0	2	5
2	4	2	2	4	2	8
2	4	2	3	10	0	13
4	4	2	0	3	13	16
2	4	2	1	12	3	16
2	6	3	1	4	13	18
2	4	2	8	6	5	19
2	4	2	2	6	0	8
2	4	2	1	7	2	10
2	5	2	0	2	6	8
2	4	2	0	0	2	2
4	9	2	1	6	6	13
2	6	2	0	4	3	7
4	10	5	5	3	9	17
2	7	2	0	1	1	2
2	4	2	0	0	2	2
2	6	2	0	9	7	16
3	7	3	6	4	26	36
2	4	2	3	10	0	13

6	9	4	1	7	4	12
3	6	3	4	5	2	11
2	6	2	0	3	3	6
2	6	2	0	4	1	5
6	19	6	2	6	3	11
2	4	2	1	0	4	5
4	6	2	3	5	2	10
2	7	2	0	3	5	8
2	4	2	0	0	3	3
2	10	2	5	3	3	11
2	4	2	2	10	6	18
3	11	5	4	2	2	8
4	8	2	8	3	11	22
3	15	2	1	3	1	5
2	4	2	1	11	10	22
2	10	4	8	5	4	17
4	8	2	4	6	12	22
6	14	5	1	0	5	6
4	6	2	2	2	0	4
6	7	3	3	6	12	21
2	4	2	0	3	3	6
2	4	2	3	0	6	9
3	12	2	10	7	13	30
2	14	2	0	4	7	11
8	17	10	9	9	11	29
2	4	2	0	1	4	5
2	4	2	3	9	11	23
4	14	4	10	6	9	25
2	5	2	5	6	13	24
2	17	5	8	13	9	30
2	4	2	1	2	4	7
4	11	2	6	3	10	19
2	4	3	3	0	3	6
2	4	2	5	4	13	22
2	4	2	0	0	3	3
6	9	2	0	7	3	10
3	13	4	4	3	15	22
5	14	2	6	6	10	22
3	8	2	0	6	3	9
2	10	4	9	6	29	44
8	20	9	9	15	21	45
2	14	2	7	6	13	26
8	11	5	4	1	9	14
3	4	2	1	9	2	12
3	11	3	2	2	4	8
3	7	2	2	2	9	13
2	6	3	4	4	3	11

2	5	2	3	2	10	15
2	4	2	4	2	12	18
5	10	3	4	4	11	19
3	6	3	0	4	11	15
2	4	2	1	0	5	6
2	15	4	0	0	15	15
2	4	2	1	4	9	14
4	13	5	6	4	12	22
5	12	2	4	13	12	29
4	6	2	0	2	5	7
2	12	3	1	7	7	15
2	4	2	5	9	9	23
4	11	4	10	11	6	27
2	7	3	3	3	26	32
4	9	3	2	3	12	17
5	11	4	2	12	2	16
2	8	4	5	11	9	25
2	11	2	6	0	3	9
3	15	4	1	1	7	9
3	12	4	1	12	7	20
2	6	3	3	3	6	12
4	12	4	3	5	6	14
2	4	2	4	0	8	12
4	16	4	5	0	2	7
4	5	2	1	0	5	6
3	8	2	2	1	6	9
2	4	2	3	8	6	17
2	6	3	2	4	7	13
4	11	2	0	6	2	8
2	9	4	4	3	21	28
4	4	2	2	6	8	16
2	10	2	3	2	0	5
2	4	2	0	0	0	0
3	5	3	3	12	20	35
3	9	3	0	0	2	2
6	13	6	4	9	8	21
2	10	3	0	10	0	10
2	8	3	5	5	0	10
3	8	2	1	4	0	5
3	9	2	4	10	5	19
3	8	2	2	11	19	32
2	4	2	1	17	23	41
2	14	2	3	11	26	40
3	11	4	0	0	3	3
2	4	2	0	7	1	8
4	15	4	3	1	9	13
3	7	3	2	5	7	14

6	10	4	0	6	2	8
6	13	8	2	12	24	38
8	16	4	2	0	4	6
3	10	2	4	15	21	40
2	4	2	8	4	27	39
2	6	4	4	6	16	26
2	7	2	5	1	15	21
5	12	4	3	3	7	13
2	9	2	1	7	1	9
2	7	2	1	3	0	4
5	9	3	6	7	16	29
4	10	3	2	6	2	10
5	9	4	3	8	11	22
3	10	5	3	3	5	11
2	6	2	3	1	17	21
3	5	3	0	2	4	6
3	10	2	2	0	1	3
2	8	2	6	2	12	20
7	20	4	4	4	8	16
2	8	2	0	5	5	10
4	8	2	2	2	1	5
2	4	2	0	1	1	2
4	8	2	0	3	7	10
5	12	5	2	6	15	23
5	6	2	3	0	16	19
8	16	3	4	4	18	26
4	14	3	3	9	13	25
5	9	4	1	3	5	9
2	4	4	2	12	10	24
2	11	2	4	6	9	19
2	4	2	0	5	4	9
3	13	5	1	6	2	9
2	4	2	1	0	0	1
3	8	3	0	13	5	18
2	6	2	0	0	0	0
2	4	2	1	1	5	7
4	6	2	1	6	7	14
2	4	2	0	2	4	6
2	10	2	0	2	3	5
4	20	7	0	3	3	6
2	4	2	0	0	1	1
2	6	3	0	1	6	7
4	9	3	1	4	2	7
5	18	2	5	3	2	10
2	6	2	5	4	3	12
2	13	2	3	3	7	13
5	15	3	3	4	12	19

5	17	3	7	3	10	20
2	11	1	0	3	0	3
2	12	2	0	0	0	0
2	4	2	2	3	0	5
3	13	2	2	7	15	24
2	8	2	0	3	1	4
2	14	3	1	0	9	10
2	14	4	10	3	14	27
4	12	2	5	4	13	22
5	12	3	2	8	3	13
2	7	4	5	7	2	14
2	12	2	6	1	18	25
2	9	4	0	6	0	6
2	5	3	2	6	8	16
6	11	2	2	4	1	7
2	4	2	4	5	15	24
6	16	6	9	12	19	40
2	8	6	0	4	1	5
3	9	2	7	2	27	36
5	9	3	4	4	14	22
2	4	2	1	4	4	9
2	7	2	0	0	8	8
2	4	2	2	2	3	7
2	8	4	0	11	4	15
2	4	2	2	13	15	27
2	4	2	0	2	6	8
2	6	2	4	0	4	8
2	16	5	2	2	5	9
4	6	4	5	5	13	23
2	4	4	3	7	9	19
3	6	3	2	2	2	6
2	10	3	6	0	14	20
2	4	2	12	4	20	36
2	4	2	3	2	4	9
2	8	4	9	3	0	12
2	4	2	11	6	13	30
2	4	2	0	4	6	10
2	7	2	3	2	2	7
2	17	3	3	10	1	14
2	6	2	0	1	3	4
4	8	2	0	0	3	3
2	4	2	6	6	3	15
5	9	2	0	3	9	12
4	8	2	2	1	1	4
2	13	4	4	6	0	10
4	18	5	8	3	13	24
5	7	5	8	9	27	44

2	6	2	3	2	14	19
2	11	2	2	2	2	6
7	10	2	1	7	0	8
2	8	3	4	1	10	15
2	4	2	3	12	19	34
2	9	4	0	12	6	18
2	6	2	0	3	1	4
2	10	2	6	1	15	22
2	4	2	0	0	0	0
6	5	4	2	4	10	16
2	4	2	1	2	20	23
4	16	5	6	0	3	9
4	9	3	4	7	22	33
2	6	2	0	10	3	13
3	5	2	1	6	8	15
2	12	4	7	8	18	33
3	8	2	2	4	3	9
3	12	2	5	0	12	17
2	5	2	4	5	12	21
3	6	4	1	1	2	4
2	8	2	4	4	1	9
2	6	2	0	3	2	5
3	4	3	1	1	25	27
2	10	3	1	1	4	6
2	10	4	1	7	1	9
2	10	2	0	6	2	8
2	7	3	0	3	5	8
3	9	2	1	0	13	14
2	8	4	2	5	3	10
4	12	5	2	8	10	20
2	8	2	3	2	1	6
2	12	4	1	5	8	14
3	9	2	2	1	7	10
3	8	2	11	3	19	33
3	5	2	0	7	5	12
2	4	2	3	2	6	11
4	11	2	5	6	0	11
2	7	4	0	4	0	4
2	12	3	0	0	2	2
2	10	2	3	7	9	19
14	6	3	0	4	3	7
4	16	5	3	11	1	15
3	8	2	0	2	4	6
3	7	2	0	5	7	12
2	4	2	1	0	0	1
4	14	4	3	1	5	9
8	18	6	4	12	27	43

2	4	2	0	4	2	6
1	10	2	3	6	11	20
3	16	4	3	5	1	9
7	13	4	11	2	4	17
2	8	3	9	13	9	31
3	9	2	6	2	16	24
2	7	2	2	2	1	5
2	8	2	0	3	0	3
2	12	2	3	4	9	16
3	13	4	2	4	17	23
3	7	2	1	2	2	5
2	6	4	0	3	10	13
2	8	2	3	4	5	12
2	10	2	4	5	22	31
2	9	6	0	8	5	13
2	6	4	6	12	1	19
3	16	7	1	15	3	19
10	20	10	6	15	16	37
2	9	2	4	11	23	38
3	9	2	1	1	1	3
5	9	2	5	0	3	8
4	9	3	1	3	5	9
4	7	3	0	0	21	21
2	13	4	5	7	4	16
3	11	3	5	9	14	28
2	13	2	0	11	3	14
6	16	6	5	3	5	13
3	11	4	9	3	13	25
2	12	2	0	4	0	4
2	7	2	2	4	3	9
2	6	5	2	1	5	8
4	10	2	4	7	14	25
2	6	3	3	10	4	17
3	6	2	0	3	3	6
2	7	3	3	6	0	9
2	4	2	6	12	17	35
2	4	2	1	8	2	11
6	15	4	3	14	23	40
3	8	2	9	3	13	25
2	8	2	0	0	0	0
2	8	5	1	0	3	4
2	6	1	0	7	4	11
2	16	2	3	7	0	10
2	6	2	4	4	1	9
3	11	3	2	6	12	20
2	10	2	4	1	13	18
3	12	3	0	7	2	9

4	6	2	1	3	7	11
2	9	2	2	4	0	6
7	20	6	9	2	18	29
2	9	2	0	0	3	3
6	17	5	3	1	6	10
2	7	3	0	0	1	1
2	6	2	3	3	1	7
2	8	2	4	2	0	6
2	12	4	0	13	3	17
4	12	4	2	2	1	5
4	12	2	4	2	2	8
5	8	3	0	0	5	5
2	4	2	2	3	0	5
3	6	3	2	2	3	7
4	13	3	8	5	18	31
3	12	4	10	2	19	31
2	9	3	5	2	6	13
2	7	2	1	4	4	9
2	10	3	3	0	0	3
2	4	2	9	3	18	30
2	7	2	7	5	9	21
2	7	4	7	12	13	32
2	8	2	0	1	12	13
2	10	3	1	9	10	20
4	7	6	1	3	1	5
2	5	2	2	4	9	15
2	9	2	0	0	2	2
2	9	2	2	7	0	9
2	7	2	1	6	9	16
4	17	6	3	10	6	19
2	14	6	6	4	0	10
4	13	1	4	7	4	15
2	7	4	5	2	5	12
3	17	4	2	8	4	14
2	12	2	0	10	3	13
3	20	8	0	5	0	5
2	4	2	3	0	2	5
3	6	3	1	2	11	14
2	6	2	7	12	25	44
3	5	3	1	0	2	3
3	6	2	0	2	5	7
3	10	4	4	5	19	28
2	4	2	0	4	2	6
2	8	4	6	12	5	23
2	6	2	1	1	2	4
2	4	2	0	6	4	10
2	4	2	1	0	9	10

10	20	6	5	9	5	19
3	9	2	0	3	0	3
2	4	2	3	3	3	9
6	17	5	3	7	0	10
2	10	2	0	2	8	10
2	8	2	0	1	4	5
4	16	5	11	3	22	36
2	12	2	2	4	11	17
2	4	2	0	0	7	7
2	10	1	5	11	14	30
2	5	2	0	3	5	8
4	7	3	2	1	9	12
2	4	2	4	1	6	11
2	8	2	6	14	14	34
2	8	3	0	4	5	9
3	10	2	0	5	5	10
2	7	2	3	4	2	9
2	15	3	2	5	3	10
2	6	2	3	6	3	12
4	12	4	3	3	7	13
8	14	8	5	7	18	30
3	8	3	4	4	9	17
2	10	3	5	9	15	29
2	7	2	0	1	11	12
5	9	2	0	7	1	8
2	7	2	1	0	2	3
3	8	3	1	6	1	8
3	11	4	1	8	4	13
2	4	2	0	5	0	5
2	13	4	4	9	20	33
3	7	2	2	10	0	12
2	8	3	8	1	16	25
2	11	5	3	5	1	9
3	9	4	4	2	8	14
6	14	7	3	5	11	19
4	19	4	0	7	0	7
2	4	2	3	10	6	19
3	12	4	1	5	18	24
3	10	2	0	2	7	9
2	4	2	0	11	3	14
2	5	2	1	8	16	25
2	12	3	5	4	1	10
2	4	2	0	5	5	10
2	6	2	0	4	1	5
2	4	2	0	6	0	6
2	4	3	3	5	0	8
5	17	6	0	3	2	5

3	16	2	7	1	17	25
2	15	2	3	6	20	29
2	17	2	4	2	7	13
6	20	8	6	6	14	26
2	4	2	2	7	6	15
2	6	2	3	5	4	12
2	4	2	3	6	3	12
2	6	2	2	4	5	11
2	6	2	9	4	15	28
3	9	2	1	5	9	15
2	4	2	0	0	2	2
2	7	2	1	3	3	7
2	4	2	0	1	0	1
2	7	2	1	0	12	13
2	4	2	7	4	16	27
3	5	2	3	12	0	15
2	4	2	2	7	0	9
2	6	2	3	5	0	8
2	5	2	0	3	0	3
2	4	2	1	1	4	6
2	4	2	0	1	3	4
2	4	2	0	0	2	2

Transtorno alimentar	IMC	Nível IMC	Atual	Desejado	Ideal Feminino	Ideal Masculino
2	14.9	1	27.5	27.5	27.5	22.5
1	18.1	1	22.5	25	22.5	27.5
2	12.9	1	17.5	20	20	22.5
2	15.3	1	22.5	22.5	30	25
2	14.0	1	15	17.5	25	25
2	16.5	1	25	25	25	20
2	28.6	3	37.5	32.5	30	32.5
2	20.8	2	22.5	20	20	27.5
2	16.8	1	25	25	25	22.5
2			25	30	30	15
2	19.5	2	25	30	30	15
2	17.1	1	27.5	22.5	20	27.5
2	32.9	4	35	30	30	32.5
1	26.9	3	25	25	20	22.5
1			32.5	30	30	25
1	29.0	3	30	22.5	22.5	27.5
2	28.7	3	35	32.5	30	27.5
1	19.8	2	20	20	27.5	20
1	20.4	2	17.5	22.5	22.5	22.5
1	14.4	1	22.5	22.5	22.5	22.5
2	20.1	2	25	25	22.5	22.5
2	21.5	2	22.5	20	17.5	
2	14.6	1	20	47.5	30	20
2	26.8	3	30	30	27.5	27.5
2	31.0	4	25	22.5	25	20
1	20.1	2	17.5	15	15	12.5
2	29.3	3	32.5	12.5	22.5	25
2	21.8	2	20	22.5	22.5	25
2	19.2	2	25	30	37.5	32.5
2	18.1	1	17.5	25	25	22.5
1	30.4	4	30	25	25	27.5
2	13.6	1	15	22.5	22.5	25
2	16.7	1	15	22.5	22.5	20
2	10.4	1	12.5	22.5	32.5	25
2	24.1	2	32.5	27	27	30
2	24.8	2	37.5	30	35	27
2	16.2	1	20	27	20	25
1	16.1	1	20	30	25	27
2	17.2	1	15	15	22.5	17.5
2	16.1	1	12.5	20		22.5
2	14.5	1	17.5	15	22.5	15
1	23.2	2	32.5	30	30	30
2	23.8	2	3			
1	34.1	4	30	25	25	22.5
2	26.2	3	30	25	25	22.5
2	17.2	1	17.5	20	30	20

2	30.8	4	22.5	22.5	35	22.5
1	22.3	2	32.5	30	27	27
2	18.0	1	20	20	25	20
1	16.3	1	20	20	22.5	20
1	18.7	2	15	15	32.5	20
2	15.9	1	12.5	20	20	15
1	23.9	2	25	22.5	22.5	20
2	18.0	1	17.5	25	25	
2	15.6	1	20	22.5	22.5	15
1	15.2	1	22.5		22.5	22.5
1	24.1	2	20	17.5	22.5	22.5
2	19.0	2	17.5	17.5	22.5	17.5
2	18.0	1	25	25	22.5	25
1	23.6	2	30	15	20	17.5
2	18.3	1	32.5		25	20
1	15.0	1	17.5	30	25	30
1	21.2	2	22.5	25	22.5	25
1	20.4	2	22.5	25	27	22.5
2	15.6	1	22.5	25	25	22.5
1	28.2	3	32.5	22.5	30	25
1	19.0	2	25	22.5	22.5	17.5
2	17.1	1	25	27	27	20
1	22.7	2			27	25
2	15.3	1	20	27	27	25
1	19.2	2	25	27	27	
2	17.8	1	20	15	25	20
2	21.9	2	20	25	27	15
2	16.6	1	17,5	17,5	25,5	0
2	22.0	2				
2	17.7	1	20	22,5	22,5	22,5
2	16.5	1	15	22,5	0	0
2	22.1	2	27,5	22,5	22,5	20
2	15.6	1	15	15	0	17,5
2	24.3	2	25	22,5	45	47,5
2	14.4	1	17,5	2,5	25	20
2	17.0	1				
2	17.0	1				
2	21.6	2				
2	19.4	2	22,5	22,5	20	22,5
2	16.4	1	12,5	15	17,5	25
2	20.3	2	17,5	17,5	22,5	17,5
2	23.7	2	40	30	25	32,5
2	25.3	3	32,5	27,7	27,5	22,5
2	22.1	2	22,5	20	30	20
2	16.3	1	20	20	25	22,5
1	26.0	3	32,5	25	220	22,5
2	18.1	1	15	22,5	0	0

2	16.2	1				
2	25.6	3	30	30	25	27,5
2	17.5	1	22,5	22,5	22,5	25
2	22.1	2	20	17,5	0	17,5
2	16.7	1				
2	16.2	1	15	15	27,5	17,5
2	16.8	1				
2	21.2	2				
2	20.9	2	22.5	22.5	22.5	20
2	17.1	1	15	22.5	20	25
2	20.8	2	20	20	25	20
2	18.0	1	20		25	20
1	23.3	2	30	25	27	22.5
2	21.5	2	30	30	30	17.5
1	16.0	1	20	22.5	22.5	17.5
2	22.9	2	30	30	30	27
1	19.5	2				
2	20.1	2	22.5	20		22.5
2	19.7	2	22.5	22.5	22.5	20
1	18.2	1	17.5	25	22.5	17.5
2	19.0	2	20	20	20	
2	16.9	1	20	20	20	
1	19.2	2	12.5	22.5	17.5	
2	16.2	1	20	25	27.5	30
1	27.5	3	32.5	22.5	22.5	30
2	17.7	1	30	27.5	27.5	30
1	22.3	2		35		32.5
1	19.5	2	22.5	25	25	17.5
1	34.1	4	37.5	27.5	30	22.5
1	21.0	2	25	22.5	20	20
2	15.0	1	20	22.5	25	20
2	32.8	4	45	30	37.5	35
2	19.7	2	22.5	27.5	25	25
1	22.6	2	25	20	22.5	17.5
2	18.7	2	17.5	17.5	17.5	20
2	18.9	2	25	27.5	27.5	17.5
1	24.0	2	32.5	22.5	27.5	12.5
1	19.6	2	30	27.5	27.5	17.5
2	18.3	1	22.5	22.5	22.5	30
1	19.3	2	17.5	25	22.5	22.5
1	29.4	3	30	25	25	27.5
1	21.8	2	20	22.5	22.5	25
2	15.3	1	30	30	30	30
2	18.5	2	12.5	12.5	20	30
2	16.8	1	20	27.5	30	17.5
2	22.1	2	22.5	20	22.5	27.5
2	18.0	1	20	22.5	20	22.5

2	26.9	3	27.5	20	20	Zero
2	200.7	6	25	25	22.5	17.5
2	20.3	2				
2	28.5	3	32.5		22.5	
2	25.5	3	32.5	30	27.5	20
2	22.9	2	22.5	17.5	20	25
2	32.3	4		35	25	
1	20.1	2	20	22.5	22.5	15
1	22.5	2	22.5	22.5	22.5	
2	22.1	2	27.5	27.5	27.5	17.5
2	17.7	1	27.5	22.5	22.5	
1	15.1	1	22.5	30	30	
1	17.4	1	17.5	20	20	17.5
1	24.9	3	30	22.5	25	25
2	36.7	5	35	27.5	25	22.5
2	14.5	1	15	22.5	20	25
1	17.9	1	20	15	20	22.5
2	16.8	1	30	32.5	25	27.5
2	29.2	3	40	25	27.5	32.5
2	16.9	1	17.5	22.5	25	20
2	19.2	2	22.5	22.5	22.5	22.5
2	16.8	1	17.5	20	17.5	25
2	20.7	2	22.5	22.5	25	15
2	59.9	6	27.5	27.5	27.5	20
2	23.7	2	25	25	22.5	22.5
2	23.7	2	27.5	25	25	22.5
2	15.0	1	35	27.5	25	22.5
2	19.5	2	25	22.5	25	22.5
2	19.8	2	20	30	25	22.5
1	24.6	2	32.5	20	20	Zero
2	30.1	4	25	22.5	25	22.5
2	17.4	1				
2	20.1	2	27.5	25	25	20
1	23.1	2	30	22.5	25	20
2	24.0	2	20	22.5	20	25
1	19.5	2	12.5	12.5	12.5	20
2	18.2	1	12.5	12.5	12.5	17.5
2	18.5	2	25	25	25	30
2	19.7	2	17.5	25	25	30
2	14.6	1	17.5	22.5	25	27.5
1	1.8	1	25	25	27.5	20
1	21.2	2	22.5	22.5	22.5	20
1	20.2	2				
2	33.0	4	20	22.5	22.5	30
2	18.1	1	25	27.5	25	30
2	25.7	3	32.5	27.5	27.5	22.5
2	26.1	3	30	32.5	35	32.5

2	22.2	2				
1	18.5	2				
2	27.8	3	35	27.5	27.5	25
1	22.3	2	15	20	17.5	20
1	16.5	1				
1	29.1	3	20	22.5	22.5	22.5
1	27.9	3	40	32.5	30	27.5
2	23.5	2	25	25	22.5	20
2	16.2	1	15	17.5	15	17.5
2	15.8	1	15		20	30
1	33.0	4				
2	19.5	2	15	15	20	20
1	19.6	2	22.5	22.5	17.5	20
2	20.5	2	25	25	25	20
1	30.0	4				
2	27.7	3	30	25	25	22.5
2	17.9	1	20	25	27.5	30
2	26.3	3	30	25	25	25
2	26.6	3	37.5	32.5	35	20
2	17.8	1	20	22.5	22.5	22.5
2	19.0	2	17.5	22.5	25	22.5
2	17.2	1	20	25	27.5	25
2	17.1	1	22.5	22.5	22.5	30
1	26.4	3	32.5	27.5	27.5	15
2	31.3	4	30	22.5	22.5	25
1	27.8	3	30	17.5	25	27.5
1	21.6	2	22.5	20	22.5	30
2	23.5	2	27.5	27.5	25	17.5
1	22.9	2	35	32.5	32.5	27.5
2	18.7	2	17.5	Zero	17.5	22.5
2	25.3	3	30	25	25	17.5
2	17.3	1	20	22.5	22.5	12.5
2	21.6	2	17.5	22.5	27.5	27.5
2	15.5	1	17.5	20	17.5	25
2	17.2	1	20	22.5	27.5	25
2	27.4	3	22.5	17.5	17.5	20
2	22.9	2	22.5	22.5	20	25
2	20.0	2	15	22.5	27.5	25
2	22.2	2	25	30	30	30
2	17.3	1	15	20	22.5	20
2	27.9	3	35	32.5	32.5	32.5
2	21.3	2	25	30	27.5	22.5
2	15.9	1	15	20	20	20
2	16.6	1	20	25	22.5	25
2	19.4	2	15	22.5	22.5	20
2	15.5	1	30	30	27.5	15
2	22.3	2	25	27.5	27.5	25

2	20.3	2	27.5	25	25	22.5
2	23.9	2	20	25	27.5	27.5
2	16.8	1	25		22.5	25
2	25.9	3	22.5	20	20	20
1	23.9	2	25	20		
2	21.9	2	27.5	27.5	27.5	20
2	30.5	4	37.5	30	32.5	22.5
1	17.3	1	17.5	22.5	22.5	20
1	18.3	1	20	20	17.5	20
2	20.7	2	22.5	27.5	25	20
2	19.6	2	22.5	22.5	32.5	27.5
1	23.5	2	20	25	25	20
2	18.4	1	27.5	20	22.5	22.5
2	19.3	2	22.5	25	25	20
2	17.1	1	17.5	20	25	25
1	23.2	2	25	20	27.5	20
1	22.6	2	17.5	22.5	27.5	30
2	24.7	2	30	20	20	25
1	25.1	3	30	22.5	25	25
1	17.6	1	17.5	22.5	27.5	30
2	23.9	2	27.5	22.5	22.5	25
2	24.6	2	35	27.5	25	25
2	22.3	2	30	22.5	22.5	25
2	17.7	1	22.5	22.5	22.5	27.5
1	20.1	2	25	20	17.5	22.5
2	18.3	1				
2	22.7	2				
2	17.6	1				
1	32.2	4	35	32.5	32.5	30
2	26.7	3				
2	18.5	2	20	25	22.5	20
2	32.0	4	37.5	27.5	22.5	22.5
1	24.7	2	22.5	20	20	20
2	22.7	2	30	17.5	22.5	20
2			15	15	15	20
1	24.4	2	30	25	25	20
2	21.4	2	25	25	25	27.5
2	17.9	1	17.5	22.5	22.5	20
2	18.9	2	15	25	27.5	22.5
2	15.7	1	17.5	20	17.5	12.5
2	17.2	1	17.5	22.5	22.5	20
2	23.0	2	27.5	27.5	27.5	27.5
2	16.0	1	20	20	20	
2	18.4	1	20	20	20	27.5
2	22.4	2	27.5	25	30	32.5
1	19.8	2	25	25	20	22.5
1	23.3	2	27.5	22.5	22.5	27.5

2	25.1	3	35	27.5	27.5	25
2	34.3	4	37.5	32.5	32.5	35
2	2.4	1	12.5	35	22.5	32.5
2	19.0	2	22.5	27.5	27.5	25
1	21.8	2	20	15	15	17.5
2	22.6	2	27.5	22.5	22.5	22.5
2	15.8	1	17.5	17.5	17.5	22.5
1	27.5	3	37.5	20	37.5	22.5
2	18.4	1	22.5	30	27.5	30
2	22.4	2	25	22.5	22.5	20
1	26.5	3	30	25	25	20
2	21.6	2	22.5	22.5	22.5	22.5
1	27.3	3	35	25	27.5	22.5
2	18.5	2	20	20	22.5	17.5
2			37.5		25	25
1	27.4	3	27.5	25	25	22.5
2	22.3	2	30	30	25	30
2	22.9	2	25	20	22.5	22.5
1	23.9	2	25	22.5	22.5	20
2	22.8	2	25	25	25	27.5
2	20.9	2	22.5	22.5	20	25
2	18.4	1	20	22.5	20	25
1	23.9	2	25	20	20	25
2	23.4	2	27.5	20	17.5	25
2	16.0	1	17.5	25	22.5	20
2	16.6	1	15	15	15	25
2	16.7	1	17.5	22.5	20	22.5
2	26.7	3	32.5	27.5	30	22.5
2	22.0	2	25	22.5	25	22.5
2	19.6	2	20	20	22.5	20
2	19.6	2	22.5		22.5	25
2	23.5	2	27.5	25	25	22.5
2	20.4	2	20	20	22.5	20
1	27.1	3	32.5	25	27.5	25
2	18.2	1	25	20	27.5	25
2	20.3	2	25	25	25	20
2			17.5	25	22.5	27.5
2	17.2	1	17.5	20	22.5	25
2	20.7	2	27.5	27.5	27.5	25
2	21.6	2	25	25	27.5	32.5
2	20.3	2	30	30	30	25
2	17.6	1	20	25	27.5	20
2	29.2	3	30	20	20	20
2	32.7	4	37.5	25	30	20
2	16.3	1	20	20	22.5	27.5
2	23.5	2	27.5	20		
1	18.9	2	25	20	22.5	17.5

2	23.7	2	30	25	27.5	25
2	32.7	4	35	25	22.5	17.5
2	17.9	1	12.5	17.5	17.5	20
2	22.4	2	25	22.5	22.5	22.5
1	17.2	1	22.5	30	35	30
1	21.1	2	25	22.5	22.5	17.5
2	25.7	3		30	25	27.5
2	18.3	1	17.5	20	20	25
2			47.5	37.5	32.5	35
1	36.0	5	37.5	27.5	22.5	30
2	18.9	2	20	25	22.5	25
2	31.1	4	27.5	27.5	20	25
2	23.4	2	27.5	27.5	27.5	25
1	23.6	2	22.5	17.5	22.5	27.5
2	12.0	1	17.5	22.5	17.5	15
2	17.5	1	20	22.5	22.5	17.5
2	18.0	1	20	22.5	22.5	17.5
1	19.8	2	32.5	25	25	25
1	24.2	2	25	20	22.5	25
2	20.0	2	27.5	25	25	22.5
2			15	15	17.5	
2			42.5	30	30	30
1	23.3	2	27.5	25	27.5	30
2	19.8	2	30	37.5	22.5	35
1	29.5	3	30	22.5	22.5	25
2	16.1	1	12.5	17.5	20	20
2	18.0	1	20	22.5	22.5	25
1	25.4	3	25	22.5	22.5	17.5
2	16.6	1	20	27.5	27.5	25
2	15.9	1	17.5	20	20	20
2	32.0	4	35	35	35	35
1	26.3	3	30	22.5	22.5	25
2	16.3	1	17.5	25	25	17.5
2	19.6	2	20	22.5	25	25
2	17.4	1	20	27.5	27.5	25
1	23.5	2	27.5	27.5	27.5	22.5
2	18.6	2	15	27.5	22.5	27.5
1	20.8	2	35		22.5	
1	21.9	2	25	27.5	27.5	20
2	19.9	2	20	22.5	25	27.5
2	21.9	2	30	30	30	27.5
2	19.2	2	17.5	25	25	17.5
2	14.6	1	15	22.5	20	20
2	17.8	1	20		20	22.5
2	24.1	2	30	22.5	22.5	20
2	22.9	2	30	27.5	25	27.5
2	17.7	1	27.5	25	30	20

2	17.9	1	22.5	22.5	27	22.5
2	18.4	1	32.5	30	30	30
1	27.8	3	25	25	25	25
2	21.5	2	22.5	22.5	22.5	20
2	19.7	2	25	25		
2	22.0	2	30		30	30
2	23.5	2	20	20	27	25
2	18.9	2	15	22.5	22.5	22.5
2	16.1	1				
2	19.4	2	22.5	22.5	35	35
2	20.1	2	27	25	22.5	25
2	24.9	3	17.5	25	27	27
2	17.4	1	25	22.5		
2	23.1	2	40	25		20
1	33.8	4	40	27	30	22.5
1	40.1	6	20	20	20	20
2	18.6	2	27	15	27	15
2	22.2	2	17.5	20	20	17.5
2	19.1	2	30	25	25	22.5
1	30.1	4				
1	19.7	2	17.5	20	22.5	25
1	17.6	1	30	22.5	22.5	20
2	23.6	2	35	20	20	20
2	20.3	2	17.5		20	27.5
2	24.5	2	22.5	22.5	22.5	30
2	26.7	3	30	30	30	
2	20.8	2	22.5	25	22.5	20
2			12.5	12.5	12.5	20
2	29.9	4	32.5	27.5		
2	19.1	2	20	20		
2	18.6	2	22.5	25	25	25
2	15.8	1	15	25	25	25
2	21.6	2	22.5	25	22.5	
2	19.3	2	20	20	25	25
2	18.8	2	22.5	25	25	25
2	18.0	1	17.5		20	25
2	23.8	2	25	25	25	
2	20.9	2	27.5	22.5	22.5	25
1	25.7	3	35	25	22.5	22.5
2	21.6	2	25	22.5	22.5	20
2	23.0	2	27.5	25	22.5	Zero
1	12.6	1	30		20	22.5
2	17.2	1	27.5	27.5	30	27.5
1	16.6	1	17.5	27.5	17.5	
2	16.9	1	17.5	15	17.5	22.5
2	16.6	1	12.5	12.5	12.5	20
2	22.2	2	27.5	25	25	25

2	21.3	2	25	27.5	25	30
2	17.9	1	15	17.5	17.5	20
2	19.7	2	15	17.5	17.5	
2	18.0	1	12.5	27.5	27.5	25
2	21.5	2	22.5	22.5	22.5	22.5
2	1.3	1	25	Zero	22.5	
1	23.3	2	30	25	25	25
2	22.4	2	25	22.5	22.5	
2	22.6	2	20	20	20	22.5
1	27.4	3	27.5	22.5	25	22.5
2	20.7	2		20	22.5	
2	40.3	6	45			
2	20.9	2	25	25	25	22.5
1	18.3	1	17.5	30	30	22.5
2	25.5	3	27.5	20	27.5	27.5
2	21.3	2	15		20	25
2	21.7	2		20	22.5	25
2	15.8	1	15		15	15
2	18.5	2	17.5	20	15	
2	21.7	2	30	27.5	27.5	15
1	25.0	3	27.5	22.5	25	22.5
2	36.0	5	17.5	17.5	22.5	20
1	25.0	3	22.5	20	20	20
2	30.2	4	30	25	25	22.5
2	17.5	1	15	22.5	22.5	22.5
2	19.8	2	20	22.5	22.5	
2	18.3	1	17.5	25	22.5	22.5
2	18.7	2	22.5	30	27.5	17.5
2	16.6	1	15	22.5	22.5	27.5
1			42.5	22.5	22.5	17.5
2			15	20	22.5	27.5
1			30	17.5		
2	17.5	1	15	27.5	25	
2	24.3	2	25	25	25	
2	24.8	2	27.5	22.5	22.5	22.5
2	16.1	1	17.5	17.5	17.5	22.5
2	17.6	1	20		25	25
1	26.4	3	27.5	22.5	22.5	22.5
2	22.2	2	20	20	20	20
2	16.2	1	15	20	17.5	
1	20.4	2	20	20	20	20
2	21.4	2	20	20	20	12.5
2	28.7	3	25	25	25	27.5
2	32.4	4	35	35	25	27.5
2	17.0	1	12.5	20	20	25
2	17.2	1	17.5	22.5	22.5	20
2	17.7	1	20	20	22.5	27.5

1	39.7	5	42.5	32.5	32.5	27.5
1	29.6	3	42.5	12.5	22.5	15
2	41.8	6	47.5	32.5	25	35
1	29.7	3	37.5	Zero	27.5	30
2	19.6	2	25	32.5	25	32.5
2	18.5	1	22.5	22.5	27.5	25
2	19.3	2	15	17.5	17.5	35
2	17.6	1	17.5	22.5	22.5	22.5
1	23.1	2	25	20	20	22.5
2	36.5	5	32.5	32.5	27.5	
2	26.2	3	37.5	35	35	27.5
2	19.8	2	17.5	17.5	17.5	25
2	18.7	2	17.5	20	22.5	
2	28.3	3	30	25	25	25
1	23.6	2	22.5	22.5	22.5	20
2	17.5	1	15	20	20	30
2	16.2	1	15	22.5	22.5	27.5
2	19.2	2	22.5	22.5	27.5	27.5
2	18.3	1	17.5	25	20	22.5
2	17.4	1	15	15	25	
2	22.3	2	22.5	22.5	22.5	25
2	20.6	2	17.5	30	30	

Perfil de participação no bullying	Ano Escolar	sexo	NSE = Nível sócioeconômico
1 = não envolvido	6º=1	1=M	A=1
2 = vítima	7º=2	2=F	B1=2
3 = agressor	8º=3		B2=3
4 = vítima-agressora	9º=4		C1=4
	1º=5		C2=5
	2º=6		
	3º=7		

Reprovação escolar	cor da pele	Transtorno alimentar	Nível IMC
reprovou=2	1 = branca	1=sim	1 = Baixo peso
não reprovou=1	2 = preta	2=não	2 = Intervalo normal
	3 = parda		3 = Sobrepeso
	4 = amarela		4 = Obesidade I
	5 = indígena		5 = Obesidade II
			6 = Obesidade III
